# Inhibition of phosphodiesterase 4 modulates cytokine induction from toll like receptor activated, but not rhinovirus infected, primary human airway smooth muscle

**DOI:** 10.1186/1465-9921-14-127

**Published:** 2013-11-15

**Authors:** David Van Ly, Monique De Pedro, Peter James, Lucy Morgan, Judith L Black, Janette K Burgess, Brian GG Oliver

**Affiliations:** 1 Woolcock Institute of Medical Research, Sydney, Australia; 2 Respiratory Research Group, Discipline of Pharmacology, The University of Sydney, Sydney, New South Wales, Australia; 3 Department of Thoracic Medicine, Concord Repatriation General Hospital, Concord, New South Wales, Australia

**Keywords:** Piclamilast, Rhinovirus, Poly I: C, Formoterol, Imiquimod, Phosphodiesterase 4, COPD, Airway smooth muscle

## Abstract

**Background:**

Virus-induced exacerbations of Chronic Obstructive Pulmonary Disease (COPD) are a significant health burden and occur even in those receiving the best current therapies. Rhinovirus (RV) infections are responsible for half of all COPD exacerbations. The mechanism by which exacerbations occur remains undefined, however it is likely to be due to virus-induced inflammation. Given that phophodiesterase 4 (PDE_4_) inhibitors have an anti-inflammatory effect in patients with COPD they present a potential therapy prior to, and during, these exacerbations.

**Methods:**

In the present study we investigated whether the PDE_4_ inhibitor piclamilast (10^-6^ M) could alter RV or viral mimetic (5 μg/mL of imiquimod or poly I:C) induced inflammation and RV replication in primary human airway smooth muscle cells (ASMC) and bronchial epithelial cells (HBEC). The mediators IL-6, IL-8, prostaglandin E_2_ and cAMP production were assayed by ELISA and RV replication was assayed by viral titration.

**Results:**

We found that in ASMCs the TLR3 agonist poly I:C induced IL-8 release was reduced while induced IL-6 release by the TLR7/8 agonist imiquimod was further increased by the presence of piclamilast. However, in RV infected ASMCs, virus replication and induced mediator release were unaltered by piclamilast, as was also found in HBECs. The novel findings of this study reveal that although PDE inhibitors may not influence RV-induced cytokine production in ASMCs and replication in either ASMCs or HBECs, they have the capacity to be anti-inflammatory during TLR activation by modulating the induction of these chemotactic cytokines.

**Conclusion:**

By extrapolating our *in vitro* findings to exacerbations of COPD *in vivo* this suggests that PDE_4_ inhibitors may have beneficial anti-inflammatory properties when patients are infected with bacteria or viruses other than RV.

## Background

Chronic Obstructive Pulmonary Disease (COPD) is characterised by irreversible airflow obstruction, inflammation and a progressive decline in lung function [1] which is increasing in prevalence and predicted to be the third leading cause of death worldwide by 2030. Exacerbations of COPD are the major cause of morbidity and mortality and are associated with accelerated decline in lung function and progression of the disease [2]. Approximately 50% of all COPD exacerbations are due to rhinovirus (RV) infections and, therefore, are a major problem for people with COPD [2,3].

The mechanism by which RV causes exacerbations is not fully understood, but is thought to be related to RV-induced activation of the innate immune system and subsequent inflammation [4]. The innate immune system detects pathogens through the expression of pattern recognition receptors (PRR), of which toll-like receptors (TLR) are perhaps the best characterized [5]. Human bronchial epithelial cells (HBEC), fibroblasts and airway smooth muscle cells (ASMC) express TLRs and are involved in the innate immune response to viral infections in the lungs [6]. During viral infections these cells can detect RV as well as other viruses through the presence of double stranded RNA (dsRNA) via TLR3, Retinoic Acid-Inducible Gene 1 (RIG-I) and Melanoma Differentiation-Associated protein 5 (MDA-5); and single stranded RNA (ssRNA) via TLR7/8 [7,8].

In the healthy airways, HBECs form the barrier between the external environment and the underlying fibroblasts and ASMCs and represent the initial point for RV infection [9]. However in COPD the bronchial epithelial layer is often compromised allowing submucosal infection to occur. Of these airway cells, ASMCs directly control bronchomotor tone and therefore make them primary drug targets for the relief of an exacerbation by β_2_ adrenoreceptor (β_2_-AR) agonists. Research has shown that RV has the ability to infect ASMCs [10,11], and ASMCs can also contribute to the local immune environment through the production of a wide variety of immunomodulatory factors such as interleukin (IL) -6 and −8 and regulating airway contraction [10,12,13]. Therefore ASMCs are undoubtedly an important contributor and direct modulator during respiratory exacerbations and investigations into these cells may offer novel therapies for exacerbations of COPD.

cAMP is a secondary signalling messenger that is able to alter the transcription of genes through the binding and activation of cAMP response element (CRE) binding protein (CREB) to CRE sequences present in the promoter region of genes [14]. IL-6 and cyclo-oxygenase (COX)-2 promoter regions both contain CRE sequences and cAMP mobilizers such as β_2_-AR agonists that induce cAMP could potentially affect these mediators [15-17]. Using IL-6 promoter constructs we have previously shown RV-induced inflammation is mediated in part via cAMP signalling [10] and others have shown that RV infection increases the activity of adenyly cyclase (the enzyme which produces cAMP) therefore sensitizing the cAMP pathway [18]. Furthermore, a synergistic increase in RV-induced IL-6 occurs in the presence of cAMP mobilizing agents such as β_2_-AR agonists [19]. Not all genes containing a CRE are pro-inflammatory (e.g. IL-10) and not all RV-induced mediators are the result of cAMP signalling. For example IL-8, which is also induced by RV *in vitro* in ASMCs, has been shown to be poorly induced in response to cAMP, suggesting its transcription may not be directly regulated by the CREB protein [20,21].

Phosphodiesterases (PDEs) regulate cAMP signalling by hydrolysis of cAMP and we have previously shown cAMP is exquisitely regulated by PDE_4_ in airway cells [22]. Inhibitors of PDE_4_ have now been developed and the PDE_4_ inhibitor roflumilast has been approved as anti-inflammatory therapy for the treatment of COPD. Roflumilast reduces sputum neutrophil (35%) and eosinophil (50%) numbers [23] and decreases the number of COPD exacerbations [8]. A similar analogue, cilomilast, has also been shown to decrease basal levels of the immunomodulatory cytokine IL-8 in HBECs obtained from patients with bronchiolitis obliterans syndrome suggesting their anti-inflammatory effects in inflammatory diseases maybe by regulating immunomodulatory cytokines via cAMP pathways directly or indirectly [24].

The main anti-inflammatory therapy used in the treatment of COPD is corticosteroids which are often used in combination with β_2_-AR agonists. Recently the efficacy of this therapy was evaluated in a large randomised control trial involving 6112 COPD patients. It was found that the use of long-acting β_2_-AR agonists and inhaled corticosteroids in combination resulted in significantly fewer exacerbations and improved health status and lung function, as compared with patients given a placebo [25]. However, corticosteroids were associated with an increased incidence of pneumonia and subsequent death, highlighting the need for better anti-inflammatory medications for the treatment of COPD.

Piclamilast is a PDE_4_ inhibitor with common structural and *in vitro* pharmacological characteristics to roflumilast [26]. We hypothesized that PDE_4_ inhibition would modulate virus-induced mediator release since many responses of ASMCs to virus infection are regulated by cAMP, and as a result decrease viral replication. The aim of this study was to investigate whether piclamilast could modulate the production of virus-induced mediators (both immunomodulatory cytokines such as IL-6 and −8 and anti-viral cytokines such as interferons (IFNs)) and virus replication in ASMCs, an important cell of the airways. Results of the study will deduce whether PDE_4_ inhibition may have potential suitability in the treatment of virus-induced exacerbations of COPD.

## Methods

### Cell culture

Primary HBECs and ASMCs were isolated from macroscopically healthy bronchial tissue obtained from patients (see Table 1 for demographics) undergoing resections or transplantations as previously described [11]. Ethical approval for all experiments involving the use of human lung tissue was provided by The University of Sydney Human Ethics Committee and the Sydney South West Area Health Service, and written informed consent was obtained. ASMCs were cultured in Dulbecco’s Modified Eagle’s Medium (DMEM) (Sigma-Aldrich, Castle Hill, Australia) supplemented with 10% (v/v) foetal bovine serum (FBS) (10% FBS/DMEM), 20U/mL penicillin, 20 g/mL streptomycin and 2.5 g/mL amphotericin B (Invitrogen, Mount Waverley, Australia) in 75 cm^2^ flasks. HBECs were cultured in selective bronchial epithelial growth medium (BEGM) (Clonetics, San Diego, California, USA) in 75 cm^2^ flasks. Cells were grown at 37°C in 5% CO_2_ until confluent at which point they were passaged further. ASMCs were identified by morphology and staining for α smooth muscle actin [27] and used for experimentation between passages 4 and 8. HBECs were used between passages 2–4. HeLa cells were maintained in 2% FBS and grown in 10% FBS supplemented in minimum essential medium (MEM), 0.025 M HEPES, 0.0375% (w/v) sodium bicarbonate and 1% GlutaMAX™ in 175 cm^2^ flasks (all from invitrogen). All cells used in this study were tested and found to be free of mycoplasma contamination.

**Table 1:** Demographic data of study patients

**Patient**	**Cell**	**Sex**	**Age**	**Disease**	**Sample**
1	ASMC	Female	27	Healthy volunteer	Bronchoscopy
2	ASMC	Female	21	Healthy volunteer	Bronchoscopy
3	ASMC	Male	56	Metastatic Melanoma	Resection
4	ASMC	Female	29	Pulmonary Hypertension	Transplant
5	ASMC	Female	43	Emphysema	Transplant
6	ASMC	Female	56	Emphysema	Transplant
7	ASMC	Female	53	Pulmonary Fibrosis	Transplant
8	ASMC	Male	66	NSCCA	Resection
9	ASMC	Male	57	Pulmonary Fibrosis	Transplant
10	ASMC	Female	59	Adeno Ca	Resection
11	ASMC	Male	75	NSCCA	Resection
12	ASMC	Female	41	Ca	Resection
13	ASMC	Female	62	Squamous Cell Ca	Resection
14	ASMC	Male	67	NSCCA	Resection
15	ASMC	Female	15	Pulmonary Hypertension	Transplant
16	ASMC	Male	58	Emphysema	Transplant
17	ASMC	Female	68	NSCCA	Resection
18	ASMC	Male	61	NSCCA	Resection
19	ASMC	Male	55	Emphysema	Transplant
20	ASMC	Male	54	NSCCA	Resection
21	ASMC	Male	67	NSCCA	Resection
22	ASMC	Male	63	NSCCA	Resection
23	ASMC	Female	68	Adeno Ca	Resection
24	ASMC	Female	60	Emphysema	Transplant
25	ASMC	Female	62	Emphysema	Transplant
26	ASMC	Female	73	NSCCA	Resection
27	ASMC	Female	57	Emphysema	Transplant
28	ASMC	Female	50	Pulmonary Fibrosis	Transplant
29	ASMC	Male	67	Emphysema	Transplant
30	ASMC	Female	78	Ca	Resection
31	ASMC	Female	51	Emphysema	Transplant
32	ASMC	Female	59	Emphysema	Transplant
33	HBEC	Female	69	Adeno Ca	Resection
34	HBEC	Female	45	Adeno Ca	Resection
35	HBEC	Male	75	NSCCA	Resection
36	HBEC	Female	58	Emphysema	Transplant

***Key: ASMC*** *airway smooth muscle cells,* ***HBEC*** *human bronchial epithelial cells,* ***NSCCA*** *non small cell carcinoma,* ***Ca*** *cancer.*

### RV propagation

Major group human RV serotype-16 was propagated in Ohio HeLa cells and purified using a 100,000 kDa molecular weight cut off (MWCO) filter as previously described [28]. RV concentrations were measured by using a tissue culture infectivity titration assay. Briefly, RV levels were determined by serially titrating log diluted concentrations of the cell free supernatant in quadruplicates on Ohio HeLa cells. Ohio HeLa cells were seeded at a concentration of 2×10^4^ cells/mL in 96 well plates (150 μL/well) and then 50 μL of supernatants with RV or control medium were added to the wells. The plates were rocked at 100 rpm for 15 minutes at room temperature before being cultured for 5 days at 37°C at 5% CO_2_. After 5 days of culture, the cytopathic effect was assessed and the tissue culture infectious dose 50 (TCID_50_) of RV was quantified using the Spearman-Karber formulation.

### Drugs

Formoterol, isoprenaline, 3-isobutyl-1-methylxanthine (IBMX), polyinosinic:polycytidylic acid (poly I:C) and imiquimod were all obtained from Sigma-Aldrich and piclamilast was obtained from Altana Pharma GmbH (Konstanz, Germany). Drugs were dissolved in dimethyl sulfoxide (DMSO) (Sigma-Aldrich) before dilution in treatment medium and vehicle controls contained the corresponding concentration of DMSO used in each experiment. Poly I:C and imiquimod were dissolved in phosphate buffer solution (PBS) (Invitrogen). The concentrations of the drugs used in this study were selected based upon previous published results [29] and/or maximal concentrations from dose response investigations.

### cAMP assay

To assess cAMP concentration, ASMCs were stimulated for 5 or 30 minutes in the presence of the phosphodiesterase inhibitor IBMX (10^-5^ M) in hank’s balanced salt solution (HBSS) (Invitrogen). In some experiments isoprenaline (10^-7^ M) was used as a positive control. The cells were then lysed in a solution of H_2_O with 0.03% (v/v) Tween-20 and 5 mM HEPES buffer by vigorous pipetting. The amount of cAMP in the lysate samples was quantified using an Alphascreen cAMP Assay Kit (Perkin Elmer, Massachusetts, USA) according to the manufacturer’s instructions and read using an EnVision Multilabel Plate Reader.

### Drug treatment and infection of primary human ASMCs with RV-16 or drugs

ASMCs or HBECs were seeded at 3.2×10^4^ cells/mL into 6 well plates in 10% FBS/DMEM and grown for 3 days. For single drug treatments, the medium was replaced with 0.1% FBS/antibiotics/DMEM or BEGM and or drug/vehicle. Alternatively, for secondary drug treatments, piclamilast was added to the medium after 1 hour of incubation with the initial drug/vehicle and incubated for 24 hours at 37°C at 5% CO_2_ prior to collection. For RV infection a cell count was carried out to determine the amount of RV needed to infect at a multiplicity of infection (MOI) of 1. Some wells were then pretreated with drug/vehicle initially for 1 hour, then infected at an MOI of 1 with live RV in that medium and left for 1 hour at 37°C and 5% CO_2_. Plates were rocked every 15 minutes to allow the virus to adsorb. The medium was removed, the cells washed with Hanks solution and 3 mL/well of 0.1% FBS/antibiotics/DMEM or BEGM supplemented with the previous drug/vehicle was added. The plates were then incubated at 37°C and 5% CO_2_ for 24 hours and supernatants were collected and stored at -80°C prior to analysis using ELISA and RV titration assays.

### ELISA

ELISA kits for the type III IFNs: IL-28B (λ_3_), IL-29 (λ_1_) and the cytokine IL-8 were purchased from R&D Systems (Minneapolis, USA); IL-6 was purchased from BD Biosciences (North Ryde, Australia); and prostaglandin E_2_ (PGE_2_) was purchased from Cayman Chemicals (Michigan, USA). ELISAs were carried out according to the manufacturer’s instructions. The detection limits of these assays were: 7.8125 pg/mL (IL-6), 15.625 pg/mL (IL-8) and 31.25 pg/mL (IL-28B (λ_3_), IL-29 (λ_1_)) and 7 pg/mL (PGE_2_).

### Statistical analysis

The data depicted in this study are presented as mean ± SEM. All data sets were verified for normality and, where they were non-parametrically distributed, the dataset was log transformed prior to statistical analysis using GraphPad Prism Version 5 software (California, USA). ELISA and RV titration results were analyzed by 1-way Analysis of Variance (ANOVA) with Bonferroni post-test comparison to their respective control and/or a paired Student's t-test where appropriate. Statistical significance was shown when p ≤ 0.05.

## Results

### Formoterol induces cAMP and IL-6 from ASMCs

**Figure 1:**
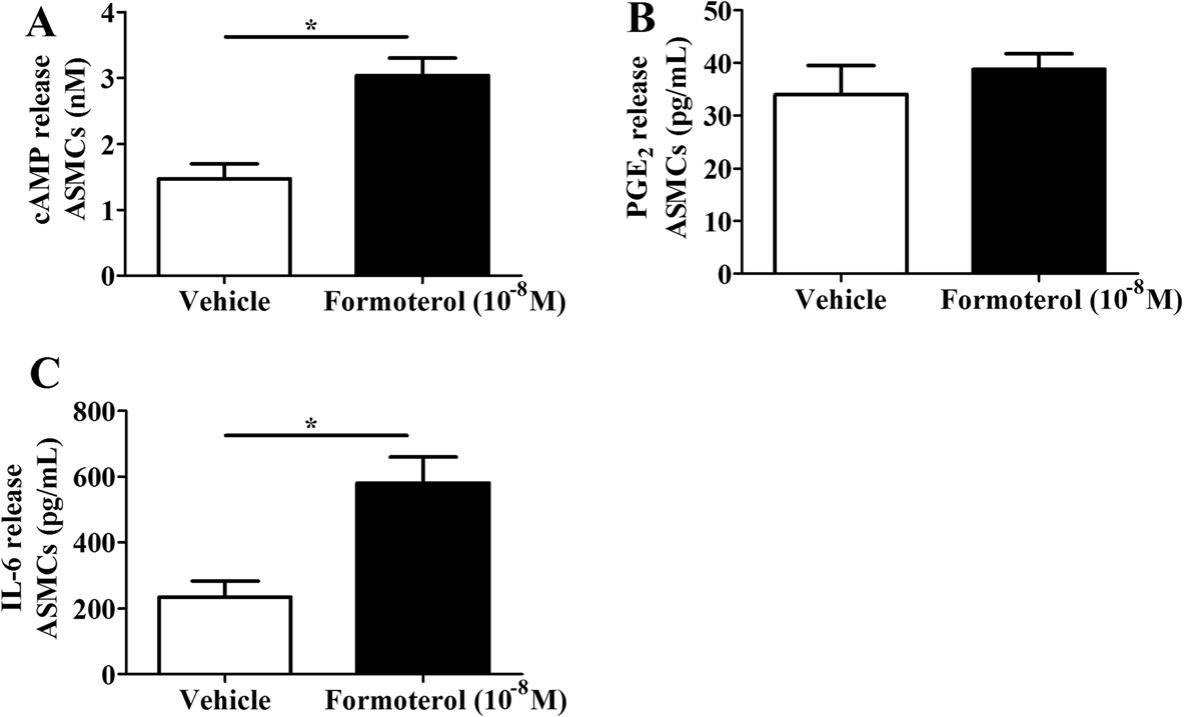
**(A-C): Effect of formoterol on cAMP, PGE**_**2 **_**and IL-6 release from ASMCs.** ASMCs were treated with medium (vehicle) or formoterol (10^-8^ M) and concentration of **A)** cAMP (n = 5) in cell lysates were measured after 5 minutes of stimulation, while **B)** PGE_2_ (n = 5) and **C)** IL-6 (n = 7) in cell supernatants were measured after 24 hrs. The vehicle control contained 10^-4^% (v/v) DMSO. *p < 0.05 between groups as indicated by the horizontal lines.

Initial experiments aimed to validate the functionality of our *in vitro* model by establishing the effects of formoterol on IL-28B, IL-29 (IFN-λ_1_&_3_), cAMP, IL-6, IL-8 and PGE_2_ production. Levels of IFN-λs were below the detection limit of the ELISA and for this reason were not investigated further (data not shown). As previously reported [29], formoterol induced cAMP and IL-6 (Figure 1A and C) but not PGE_2_ compared to vehicle controls (Figure 1B) in ASMCs. The level of IL-8 assessed in this experiment was below the detection limit of the ELISA (data not shown).

### Inhibition of PDE_4_ increased formoterol-induced cAMP, basal cAMP and IL-6 from ASMCs

**Figure 2:**
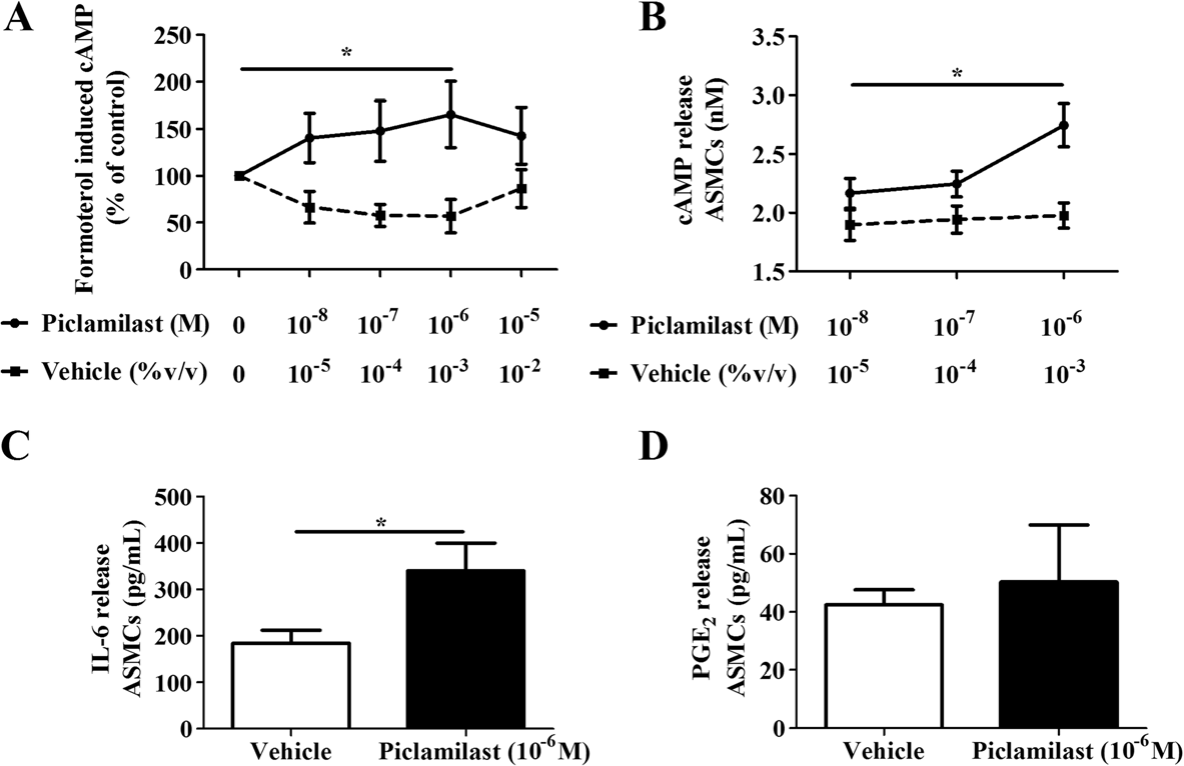
**(A-D): Effect of piclamilast on: formoterol-induced cAMP, accumulated cAMP, IL-6 and PGE**_**2 **_**release from ASMCs.** Concentration of **A)** formoterol-induced cAMP (n = 6) in cell lysates were measured after 1 hr pre-treatment with piclamilast (10^-8^-10^-5^ M) or corresponding vehicle (10^-5^-10^-2^% (v/v) DMSO) followed by 5 minutes of stimulation with formoterol at 10^-8^ M. Concentration of **B)** accumulated cAMP (n = 5) in cell lysates were measured after 24 hr pre-treatment with piclamilast (10^-8^-10^-6^ M) or corresponding vehicle (10^-5^-10^-3^% (v/v) DMSO). Concentrations of **C)** IL-6 (n = 17) and **D)** PGE_2_ (n = 4) in cell supernatants were collected at 24 hrs from ASMCs treated with vehicle (10^-3^% (v/v) DMSO) or piclamilast (10^-6^ M). *p < 0.05 between groups is compared to the control or the least concentration of piclamilast as indicated by the horizontal lines.

**Figure 3:**
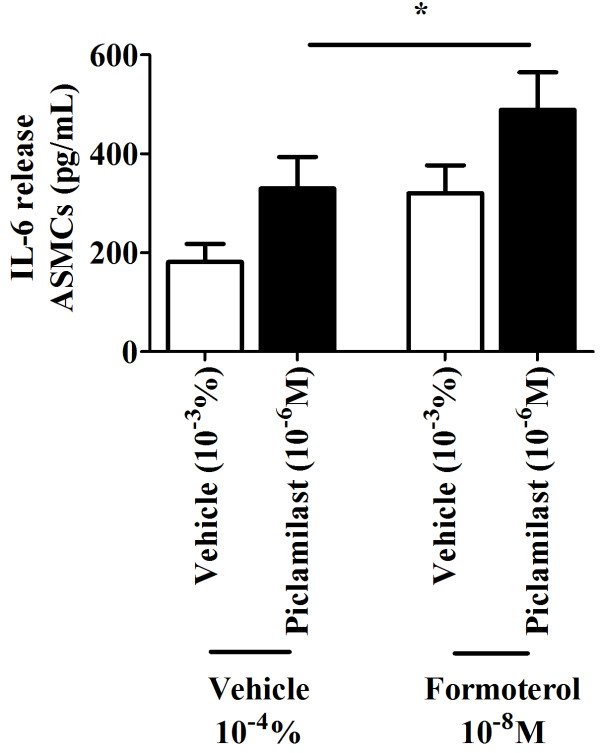
**Effect of piclamilast on formoterol-induced IL-6 from ASMCs.** Concentrations of IL-6 (n = 13) in cell supernatants were collected at 24 hrs from ASMCs treated with corresponding vehicle (10^-3^% (v/v) DMSO) or piclamilast (10^-6^ M) in the presence of formoterol (10^-8^ M) or the corresponding formoterol vehicle (10^-4^% (v/v) DMSO). *p < 0.05 between groups as indicated by the horizontal lines.

The functional activity of piclamilast was confirmed by showing that inhibition of PDE_4_ further increased formoterol-induced cAMP as well as increasing the basal accumulation of cAMP over 24 hours (Figure 2A & B). Given that the functional activity of piclamilast was most effective at a concentration of 10^-6^ M this concentration was chosen for subsequent experiments. Piclamilast induced IL-6 release from ASMCs compared to vehicle treatment but did not influence PGE_2_ release (Figure 2C & D). The level of IL-8 assessed in this experiment was below the detection limit of the ELISA (data not shown). As shown in Figure 3, IL-6 production is additively increased when ASMCs were treated with piclamilast in the presence of formoterol at 10^-8^ M (Figure 3).

### PDE_4_ inhibition modulates TLR agonist-induced cytokine release from ASMCs

**Figure 4:**
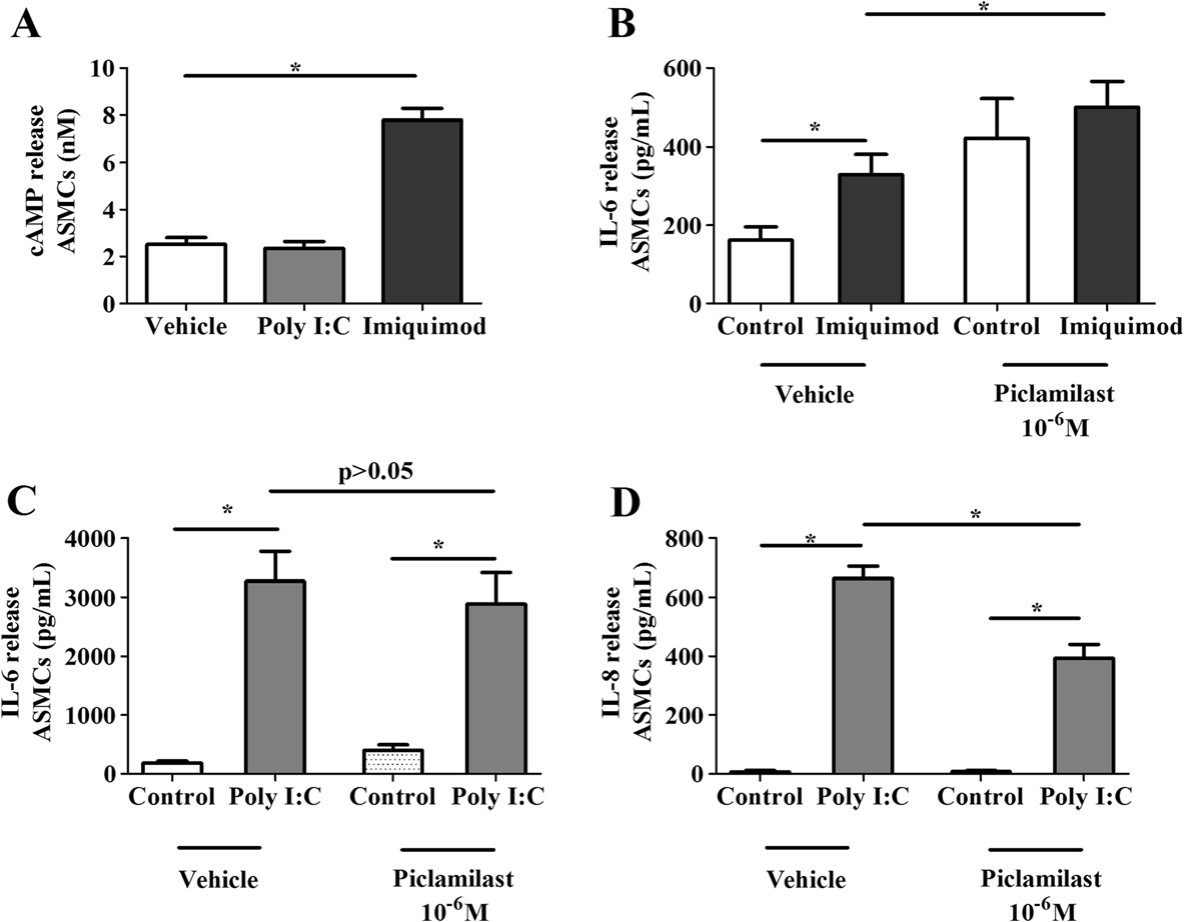
**(A-D): Effect of imiquimod and poly I:C on cAMP, IL-6 and IL-8 release from ASMCs in the presence or absence of piclamilast.** Concentration of **A)** cAMP (n = 6) in cell lysates were measured after 30 minutes of stimulation with imiquimod (50 μg/mL) and poly I:C (50 μg/mL). Concentrations of IL-6 **(B, C)** and IL-8 **(D)** in cell supernatants were collected at 24 hrs from ASMCs treated with control (vehicle); imiquimod (5 μg/mL) (n = 10) and poly I:C (5 μg/mL) (n = 9) in the presence of vehicle or piclamilast (10^-6^ M). The vehicle control consisted of 10^-3^% (v/v) DMSO. *p < 0.05 between groups as indicated by the horizontal lines.

The agonists for TLR 3 and 7/8: poly I:C and imiquimod respectively (surrogates for double stranded and single stranded RNA) were used to treat ASMCs in order to investigate the effects of PDE_4_ inhibition on the sequelae of TLR activation. To investigate if the TLR agonists directly modulated cAMP we measured cAMP from ASMCs stimulated with TLR agonists for only 30 minutes, to avoid the confounding effects of autocrine action of mediators such as prostaglandins [11]. We found imiquimod induced cAMP compared to vehicle control but poly I:C did not (Figure 4A). Imiquimod did not induce IL-8 (below detection limit of the ELISA) (data not shown) but induced IL-6 from ASMCs compared to vehicle control and this was additively increased in the presence of piclamilast (Figure 4B). In comparison, poly I:C induced both IL-6 and IL-8 in ASMCs compared to vehicle control but piclamilast inhibited poly I:C-induced IL-8, and had no effect on IL-6 (Figure 4C & D).

### PDE_4_ inhibitors do not modulate RV-induced cytokines and RV does not induce cAMP

**Figure 5:**
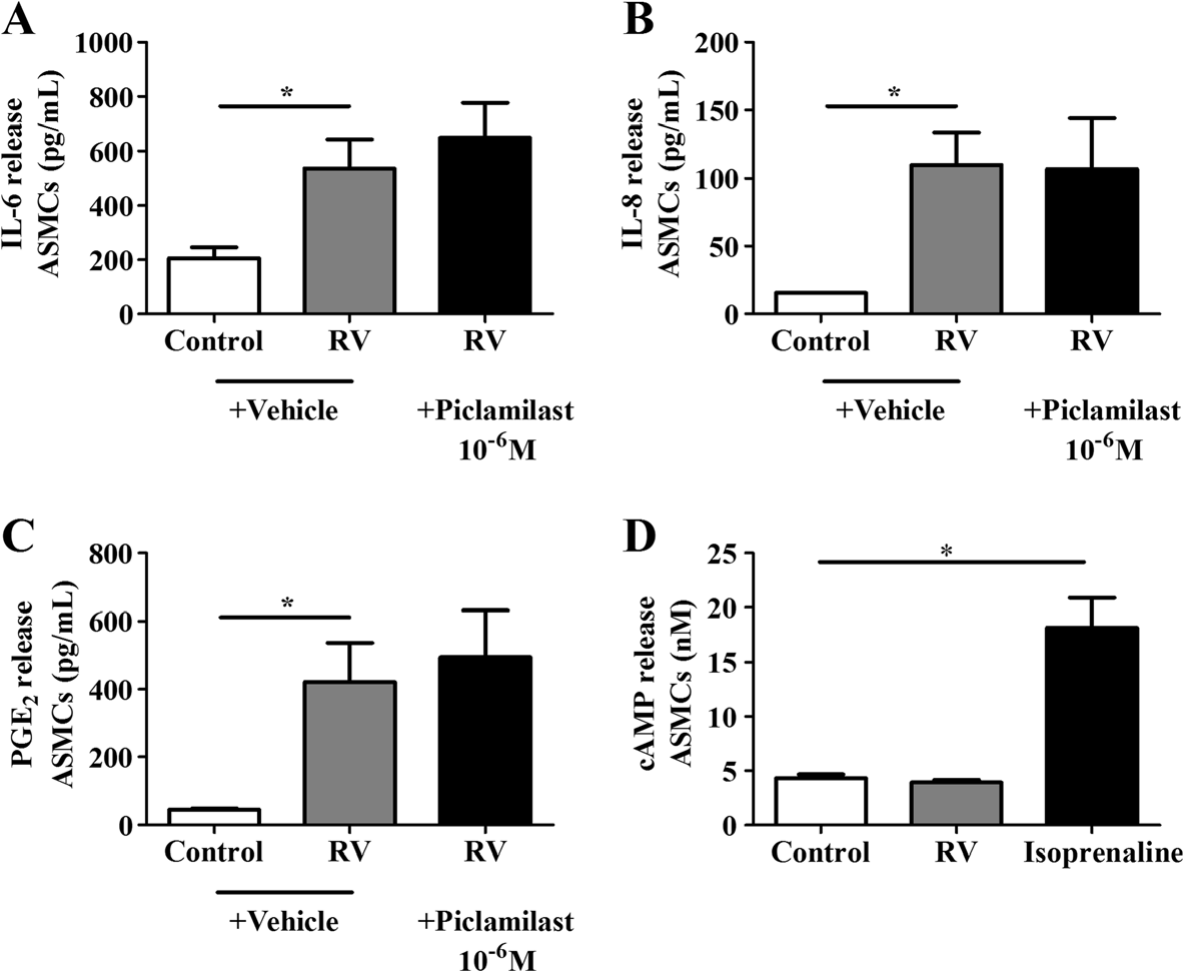
**(A-D): Effect of RV-16 on IL-6, IL-8 and PGE**_**2 **_**release from ASMCs in the presence or absence of piclamilast.** Concentrations of **A)** IL-6 (n = 7), **B)** IL-8 (n = 7) and **C)** PGE_2_ (n = 4) in cell supernatants were collected at 24 hrs from ASMCs treated with control and RV (MOI = 1) in the presence of vehicle or piclamilast (10^-6^ M). Concentration of **D)** cAMP (n = 6) in cell lysates were measured after 30 minutes of infection (MOI = 1) or stimulation with the positive control isoprenaline (10^-7^ M). The vehicle controls contained 10^-3^% (v/v) DMSO. *p < 0.05 between groups as indicated by the horizontal lines.

In order to assess the effects of PDE_4_ inhibition on viral induced inflammation ASMCs were infected with purified live RV-16. RV infection of ASMCs significantly increased the induction of IL-6, IL-8 and PGE_2_ compared to constitutive release in the presence of vehicle but piclamilast did not alter RV induction of those cytokines (Figure 5A-C). We also found that piclamilast did not alter RV-induced IL-6 (41.2 ± 17.3 pg/mL vs 59.4 ± 23.0 pg/mL, n = 4 RV verses RV + piclamilast) and IL-8 (437.0 ± 163.5 pg/mL vs 531.5 ± 204.1 pg/mL, n = 4 RV verses RV + piclamilast) in HBECs. Furthermore, RV did not induce cAMP within 30 minutes of infection compared to uninfected cells (Figure 5D).

### Inhibition of PDE_4_ does not modulate RV replication in ASMCs or HBECs

**Figure 6:**
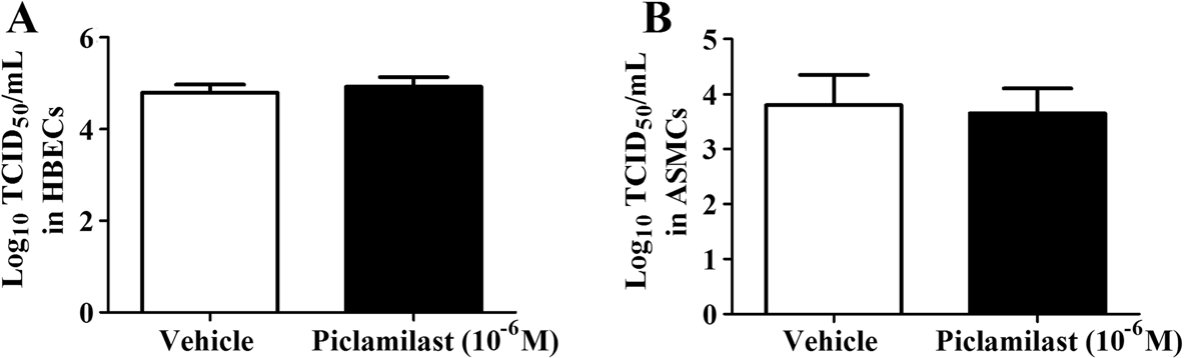
**(A-B): Piclamilast did not modulate RV replication in HBECs and ASMCs. A)** HBECs (n = 4) and **B)** ASMCs (n = 5) were infected with RV at an MOI = 1 in the presence of vehicle (10^-3^% (v/v) DMSO) or piclamilast (10^-6^ M) and RV concentration in cell supernatants were quantified after 24 hours.

Subsequently we investigated if inhibition of PDE_4_ could result in changes to RV replication in ASMCs and in HBECs as HBECs are a source of antiviral IFNs which may interfere with RV replication. Piclamilast did not alter the virion number in either cell type (Figure 6A & B).

## Discussion

Virus-induced exacerbations of COPD constitute a significant health burden and its management is hampered by the lack of effective therapies. The mechanism by which exacerbations occur remains undefined however it is likely due to virus-induced inflammation. Given that PDE_4_ inhibitors have been clinically shown to have anti-inflammatory effects in patients with COPD they present a potential therapy during these exacerbations. In the present study we investigated whether the PDE_4_ inhibitor piclamilast could alter virus-induced cytokine release and replication. The novel findings of this study reveal that although PDE_4_ inhibitors may not influence RV-induced cytokine release and replication in ASMCs, a potential mechanism by which PDE_4_ inhibitors may be anti-inflammatory could be via the increase of IL-6 and inhibition of IL-8 induction, resulting in reduced neutrophil numbers under inflammatory conditions.

In the present study we initially established that IL-6 induction is regulated by cAMP by showing that inducing cAMP using formoterol or inhibiting cAMP hydrolysis using piclamilast positively induces IL-6 and cAMP, but not IL-8 or PGE_2,_ from ASMCs. Although the findings with IL-6 induction are consistent with others [16] we did not observe any effects on IL-8 release from ASMCs. Pang *et al.* showed that the β_2_-AR agonists salbutamol and salmeterol induced IL-8 release from ASMCs at concentrations greater than 10^-6^ M, which were at least 2 logs greater than those used in the present study [20]. At such high concentrations it is possible that transcription factors other than CREB could be activated [30] suggesting that at least in ASMCs, IL-8 transcription may not involve cAMP but rather cross talk between other transcription factors. Furthermore, the lack of change in PGE_2_ levels may be the result of being dominantly regulated by alternative signalling pathways such as NF-*κ*B [31]. Since PDEs regulate cAMP signalling which in turn can regulate immunomodulatory cytokines, we then investigated whether manipulation of this pathway with the PDE_4_ inhibitor piclamilast could result in antiviral or anti-inflammatory effects on ASMCs during viral-induced inflammation.

Virus replication generally involves the transcription of viral genomic material to produce new viral progeny. The cells of the innate immune system have evolved to detect foreign viral particles through TLRs. Activation of TLRs result in the increase of COX-2 induced PGE_2_ which subsequently increase cAMP levels and leads to increased IL-6 and IL-8 [11,32,33]. Therefore, in order to assess whether piclamilast could modulate virus-induced inflammation via TLR activation, analogues of TLRs such as poly I:C and imiquimod were used to stimulate TLRs 3 and 7/8 respectively on ASMCs.

Imiquimod only induced cAMP and IL-6 release from ASMCs and the addition of piclamilast increased levels of IL-6, most likely by extending cAMP activity, similar to those effects of formoterol and piclamilast. Interestingly, poly I:C induced both IL-6 and IL-8 release from ASMCs but did not induce cAMP. This suggests that both cytokines may also be regulated by signalling mechanisms other than cAMP, such as NF-κB and MAPK signalling [34]. On the other hand piclamilast reduced poly I:C-induced IL-8 release which was similar to the findings by Murphy *et al.* using cilomilast, a PDE4 inhibitor, on HBECs obtained from patients with bronchiolitis obliterans syndrome [24]. Unlike corticosteroids, the sequelae of increasing cAMP is not pan inhibition of cytokine release, for example in ASMCs others have shown that some but not all IL-1β-induced cytokines are inhibited as a consequence of stimulating cAMP production [35]. We speculate the mechanism behind inhibition of poly I:C-induced IL-8 by PDE_4_ inhibition might be via cAMP sequestration of the NF-κB pathway downstream of immuno-receptors as was shown to be true in splenic B lymphocytes [36]. IL-6 is a cytokine with multiple biological roles and traditionally considered a pro-inflammatory marker as its levels are often detected to be increased in inflammatory diseases such as asthma [37]. However the biological functions of IL-6 do not make it entirely a contributor to inflammation as more recently other roles which contribute to the resolution of the innate immune response, such as reducing neutrophil trafficking during acute inflammation, have been uncovered [38]. Since reports have shown that IL-6 can limit the recruitment of neutrophils and oppositely, IL-8 can induce neutrophil chemotaxis [38,39], our results suggest that the increase in IL-6 and reduction of IL-8 cytokine release by PDE_4_ inhibition during inflammatory circumstances may be important in the anti-inflammatory mechanism of action of PDE_4_ inhibitors.

In order to assess the effects of PDE_4_ inhibition on virus infection, as opposed to the use of viral surrogates, ASMCs were infected with RV-16. RV infection of ASMCs resulted in the induction of inflammatory cytokines and lipids including IL6, IL-8 and PGE_2_ but were unaffected by PDE_4_ inhibition. Similarly, RV infection of HBECs which have been shown by others to induce IL-6 and IL-8 [40] were unaffected by PDE_4_ inhibition. Significantly our study also shows that RV infection in ASMCs did not induce cAMP in an acute setting. In a longer incubation, RV may be able to induce cAMP through its replication, as increased viral replication will result in the activation of TLRs and subsequently the induction of cAMP and cytokines by COX-2 induced prostaglandins [11]. Since RV infection causes sensitization of adenylyl cyclase [18] these prostaglandins can further induce cAMP induction in an autocrine manner as we have previously shown [11]. Although we initially established that cAMP induction correlates to increased levels of IL-6 in our study there are limitations that should be noted. Because cAMP signalling occurs earlier than protein production, it would be interesting to quantify the amount of cAMP that is required to induce a certain amount of IL-6 at 24 hours; however this will be difficult in the context of RV infection. The reason for this being measurements of cAMP over 24 hours will be confounded by factors such as RV-induced cell death and the influence of other induced mediators such as prostaglandins. Nevertheless, these results suggest that RV-induced inflammation is complex, with the activation of multiple inflammatory signal pathways such as NF-κB, cAMP signalling and prostaglandins pathways, as TLRs are activated during increased RV replication [10,19,41].

RV infections are major contributors to exacerbations of COPD [42,43]. Methods to prevent or treat these infections could aid in reducing COPD exacerbations, however there are currently no anti-rhinoviral pharmacotherapies on the market. Anti-inflammatory corticosteroids are already widely used in the treatment of airway inflammation. They have demonstrated inhibitory effects on RV replication in epithelial cell culture possibly by reducing the expression of the RV entry receptor ICAM-1 [44,45]. However this effect appears to be cell dependent as corticosteroids do not alter RV replication in cultured airway fibroblasts [41]. Clinically, corticosteroids do not appear to have an effect on the symptoms of the common cold and lead to prolonged RV replication in the airways [46]. Therefore, improved medications are needed for both anti-inflammatory and anti-viral treatments in exacerbations of COPD and asthma.

PDE_4_ inhibitors inhibit TNF-α stimulated expression of ICAM-1 in fibroblasts suggesting that, as with corticosteroids, they may be able to inhibit RV infection by reducing ICAM-1 expression [47]. The present study showed that the PDE_4_ inhibitor piclamilast had no effect on RV replication, demonstrating that either ASMCs do not have the ability to inhibit RV replication or that cAMP signalling does not regulate the innate anti-viral response to RV. IFNs are a major anti-viral group of cytokines and, in support of the former possibility, levels of IFN-λ measured in cell supernatants of infected ASMCs in this study were below the detection limit of the ELISA. Although Calven *et al.* showed that infection with RV-1B induced significant mRNA levels of the type I IFNs (IFN-β) and IFN-λ_1_in bronchial ASM, their corresponding protein levels post-infection in cell supernatants were reported only just at detection level. Similarly, others have shown that primary human ASMCs and lung fibroblasts do not produce IFNs in response to RV infection [10,41,48]. In this regard, the biological significance of the level of induced IFN proteins from ASMCs and, whether ASMCs have the ability to produce IFN proteins, remains controversial. If ASMCs do not produce IFNs then it is highly likely that they do not possess the ability to inhibit RV replication in the absence of other cell types. Bronchial epithelial cells on the other hand express and produce IFNs in response to RV infection, and in a multicellular environment may have the ability to induce antiviral effects on other cells such as ASMCs and fibroblasts [49,50]. However, in this study piclamilast also had no effect on RV replication in HBECs, highlighting the fact that cAMP signalling may not be regulating the innate anti-viral response to RV, or a more complex system underlies regulation of viral replication than just IFNs.

There is a vast amount of evidence to suggest that cells from different disease states respond and behave differently to each other. For example asthmatic ASMCs and epithelial cells produce more IL-6 and less IFNs respectively in response to RV infection compared to non asthmatic cells [10,49]. In this study we examined the effects of PDE_4_ inhibitors on viral replication and inflammation in a pooled group of primary human ASMCs obtained from various patients with respiratory diseases. Since there were insufficient cells for each disease group we were not able to compare cell responses between diseased populations. Therefore future studies looking at the differences between healthy and COPD or asthmatic cells may further reveal whether the role of PDE_4_ during RV, as well as other respiratory viral infections, is altered in different diseases.

Additionally, in our study we showed that ASMCs produce levels of IL-6 in response to formoterol which is further increased by PDE_4_ inhibition. Although this is consistent with the findings by other *in vitro* studies [29] intuitively this would suggest that β_2_-AR agonists create a pro-inflammatory environment which could be detrimental to the inflammatory conditions already present in diseases such as COPD. However this is not the case, because in *in vivo* mouse model studies of inflammatory lung disorders, β_2_-AR agonists have been shown to have anti-inflammatory effects and reduce IL-6 levels [51,52]. While clinically, β_2_-AR agonists have been shown to be very effective therapies in inflammatory respiratory diseases such as asthma when combined with corticosteroids [53]. Why *in vivo* and *in vitro* responses differ is not known however it is likely due to the combined multicellular interaction and response to β_2_-AR agonists, thus highlighting a potential limitation of our monocellular study. In tissue where all cells are present, it is likely that the pool of mediators will complement each other in a physiological interaction. For this reason it is unlikely that physiologically any single group of cells would be more or less important than another in terms of cytokine induction. Therefore future studies could expand on this study using *in vitro* co-culture systems and/or *in vivo* mouse models to better demonstrate a more multicellular interactive response to β_2_-AR agonists and PDE_4_ inhibitors.

## Conclusion

A characteristic feature of COPD is inflammation of the lungs with increased levels of IL-8 detected in patient bronchoalveolar fluids compared to non COPD patients [54]. In COPD patients PDE_4_ inhibitors have been shown to be anti-inflammatory by reducing neutrophils and eosinophils in their sputum [23]. Others have also shown that PDE_4_ inhibitors can reduce basal inflammatory levels of IL-8 in HBECs obtained from patients with the severe inflammatory respiratory disorder: bronchiolitis obliterans syndrome [24]. Our study suggests that this reduction of neutrophils may be due to the ability of PDE_4_ inhibitors to modulate IL-6 and also IL-8 levels in ASMCs and therefore limit neutrophil recruitment [38]. PDE_4_ inhibitors have been shown to synergistically increase the effects of β_2_-AR agonists, and dual specific PDE_4/3_ inhibitors have been shown to cause acute short acting bronchodilation in people with asthma [55-57]. We demonstrated for the first time that PDE_4_ inhibitors do not alter RV replication or the inflammatory response to RV infections in ASMCs and HBECs. Because PDE_4_ inhibitors do not modulate RV-induced inflammation or replication this study suggests PDE_4_ inhibitors may be safe to supplement bronchodilating medication during RV-induced exacerbations of COPD and asthma as well as having other beneficial effects of reducing inflammatory neutrophils during exacerbations of COPD caused by bacteria or viruses other than RV.

## Competing interests

The authors declare that they have no competing interests.

## Authors’ contributions

DV and BGGO designed these studies with contribution from LM, JLB and JKB; DV, MD and PJ carried out the experiments, analyzed the data and interpreted the results of the experiments; DV prepared and drafted the manuscript; DV, JLB, JKB, LM and BGGO edited, revised and approved the final version of the manuscript.
